# Identifying protein-protein interface via a novel multi-scale local sequence and structural representation

**DOI:** 10.1186/s12859-019-3048-2

**Published:** 2019-12-24

**Authors:** Fei Guo, Quan Zou, Guang Yang, Dan Wang, Jijun Tang, Junhai Xu

**Affiliations:** 1 College of Intelligence and Computing, Tianjin University, Tianjin, People’s Republic of China; 2 Institute of Fundamental and Frontier Sciences, University of Electronic Science and Technology of China, Chengdu, People’s Republic of China; 3 School of Economics, Nankai University, Tianjin, People’s Republic of China; 4 Department of Computer Science, City University of Hong Kong, Kowloon Tong, Hong Kong; 5 Department of Computer Science and Engineering, University of South Carolina, Columbia, USA

**Keywords:** Protein-protein interface, Multi-scale local average block, Hexagon structure construction

## Abstract

**Background:**

Protein-protein interaction plays a key role in a multitude of biological processes, such as signal transduction, de novo drug design, immune responses, and enzymatic activities. Gaining insights of various binding abilities can deepen our understanding of the interaction. It is of great interest to understand how proteins in a complex interact with each other. Many efficient methods have been developed for identifying protein-protein interface.

**Results:**

In this paper, we obtain the local information on protein-protein interface, through multi-scale local average block and hexagon structure construction. Given a pair of proteins, we use a trained support vector regression (SVR) model to select best configurations. On Benchmark v4.0, our method achieves average *I*_*rmsd*_ value of 3.28Å and overall *F*_*nat*_ value of 63*%*, which improves upon *I*_*rmsd*_ of 3.89Å and *F*_*nat*_ of 49*%* for ZRANK, and *I*_*rmsd*_ of 3.99Å and *F*_*nat*_ of 46*%* for ClusPro. On CAPRI targets, our method achieves average *I*_*rmsd*_ value of 3.45Å and overall *F*_*nat*_ value of 46*%*, which improves upon *I*_*rmsd*_ of 4.18Å and *F*_*nat*_ of 40*%* for ZRANK, and *I*_*rmsd*_ of 5.12Å and *F*_*nat*_ of 32*%* for ClusPro. The success rates by our method, FRODOCK 2.0, InterEvDock and SnapDock on Benchmark v4.0 are 41.5*%*, 29.0*%*, 29.4*%* and 37.0*%*, respectively.

**Conclusion:**

Experiments show that our method performs better than some state-of-the-art methods, based on the prediction quality improved in terms of CAPRI evaluation criteria. All these results demonstrate that our method is a valuable technological tool for identifying protein-protein interface.

## Background

In biological processes, many proteins carry out the special biological functions through protein-protein interactions, such as drug design and functional analysis. Gaining insights of various binding abilities can deepen our understanding on protein-protein interface. Determination of binding sites is widely applied in molecular biology research. It is of great interest to understand how proteins bind with each other, which helps us understand energetics and mechanisms of complexes. How to build more effective models based on sequence information, structure information and physicochemical characteristics, is the key technology for identifying protein-protein interface. There are many efficient techniques for the protein-protein interface prediction [1–11].

Some approaches use machine learning methods and statistical methods to analyze the differences between interface residues and non-interface residues on the surfaces [12–15]. ProMate [16] creates the circle around each surface residue, which can be used to extract the statistical histogram of many features. Then, it estimates the probability of each circle to be on the interface, and some circles with high probability values are clustered to identify binding residues. PPI-Pred [17] generates an interacting patch and a non-interacting patch for each training protein, and extract several features from these patches to build an SVM model for predicting the interacting patch in each testing protein. PINUP [18] proposes an empirical scoring function, including interface propensity and residue conservation score. It calculates the occurrence of each top scoring spot, therefore predicts residues on interface spots. Meta-servers combine the strengths of some existing approaches: meta-PPISP [19] combines three prediction servers; metaPPI [20] combines five identification methods. ProBiS [21, 22] predicts protein-protein interface by local structure alignment. It compares the information of a testing protein to some binding sites in the known database, for detecting similar structural residues.

Another kind of methods check the possible poses of two subunits; that is, how these subunits may dock. Docking methods based on fast Fourier transformation (FFT) [23], geometric surface matching [24], as well as intermolecular energy [25] have been proposed. The general approach is to explore all possible poses, and use one energy function to identify near-native poses. The problem of exploring all possible poses has been well-solved by some methods [26–28]. The key issue here is to design an energy function based on various properties and features that can identify near-native poses, such as hydrophobic and conserved polar at specific locations [29], hydrogen bonds and salt bridges [30], secondary structure composition [31], relative surface area burial and weighted hydrophobicity [32], force field energy evaluation [33–35]. FRODOCK 2.0 [36] presents an user-friendly protein-protein docking server based on an improved version including a complementary knowledge-based potential. InterEvDock [37] is a server for protein docking based on a free rigid-body docking strategy, intergrating co-evolutionary information. SnapDock [38] is a highly efficient template-based protein-protein docking algorithm, utilizing the interface PIFACE library. CIPS [39] proposes a new pair potential combining interface composition with residue-residue contact preference, screening docking solutions obtained either with all-atom or with coarse-grain rigid docking. ZRANK [40, 41] combines an atom-based potential (IFACE) with five residue-based potentials for ranking solutions. It provides fast and accurate re-scoring models from ZDOCK. ClusPro [42] develops a fast algorithm for filtering docked conformations with good surface complementarity and ranking them based on their clustering properties. RosettaDock [43] constructs the energy function by using van der Waals energies, orientation-dependent hydrogen bonding, implicit Gaussian solvation, side-chain rotamer probabilities and a low-weighted electrostatics energy. HADDOCK [44] makes use of the biochemical and biophysical interaction data, such as chemical shift perturbation data resulting from NMR titration experiments.

In this paper, we calculate the local information on the protein-protein interface, through multi-scale local average block and hexagon structure construction. Given a pair of input proteins, we use the trained support vector regression (SVR) model to select best protein-protein docking poses. Experiments show that our method achieves better results than some state-of-the-art methods. Here, we use the CAPRI evaluation criteria [45], *I*_*rmsd*_ value and *F*_*nat*_ value. On Benchmark v4.0 [46], our method has average *I*_*rmsd*_ value of 3.28Å and overall *F*_*nat*_ value of 63*%*. On the CAPRI targets, our method has average *I*_*rmsd*_ value of 3.45Å and overall *F*_*nat*_ value of 46*%*. The success rates by our method on Benchmark v4.0 are 41.5*%*. Comparing to the existing methods, our method is a valuable technological tool for identifying protein-protein interface.

## Methods

We find the relative orientation and position between two subunits, and each relative orientation and position combination is referred to as a configuration or pose. Given a configuration, we can determine the interface region between two subunits and fix the orientation as well as position of the regions far from the interface.

**Fig. 1:**
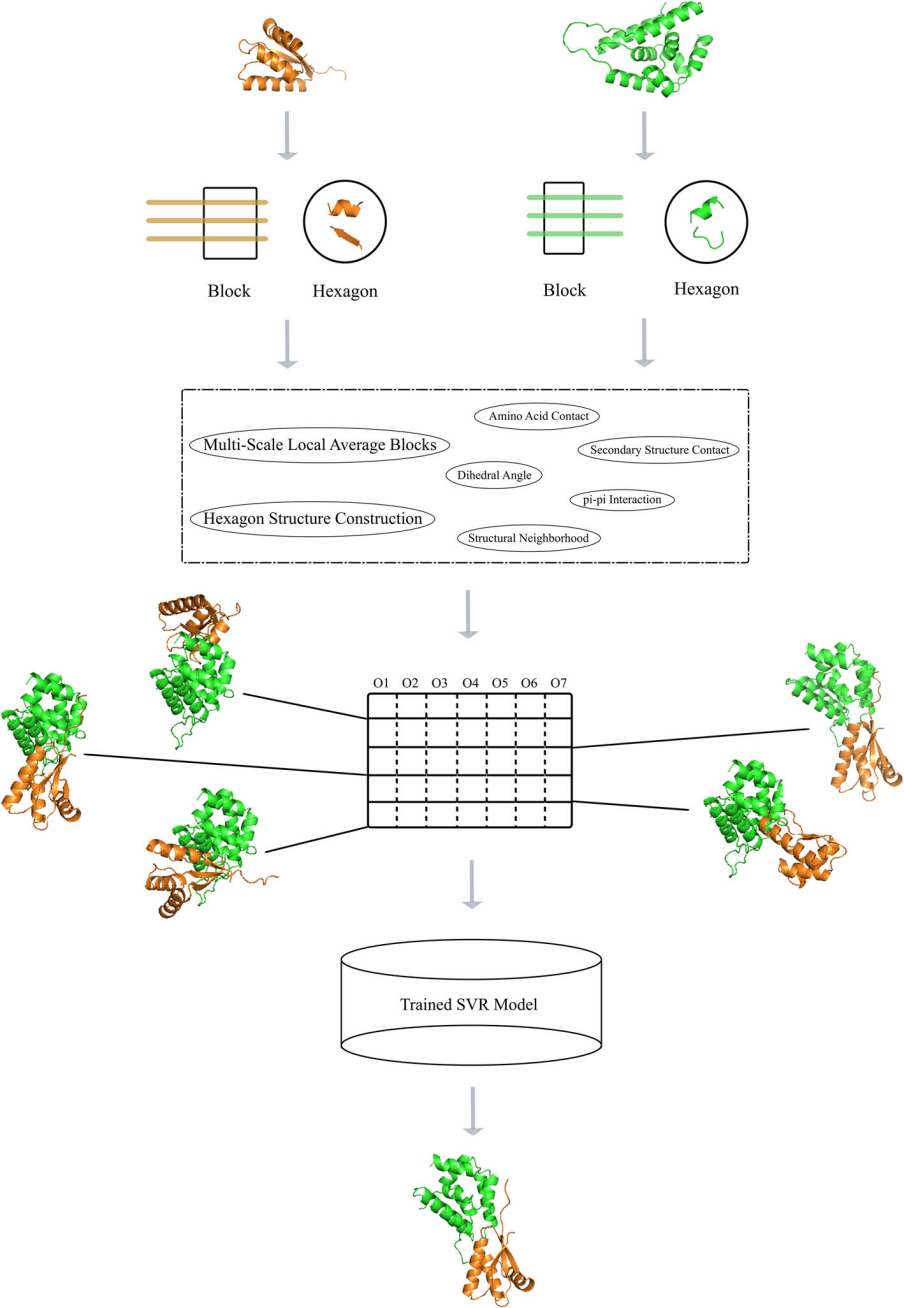
The flowchart of our method for identifying protein-protein interface

Here, we utilize our previous enumeration method [47] to identify the docking configurations of two subunits. It performs a large number of rigid transformations to enumerate the poses. Then, we design a novel energy function and build a trained SVR model to evaluate docking poses and select the top-ranking poses with lowest energy values. The flowchart is shown in Fig. 1.

In this paper, our main work is to obtain the local information on protein-protein interface for energy evaluation. First, each pair of proteins can be encoded with physicochemical property and position specific scoring matrix. Then, we establish two novel models, multi-scale local average block and hexagon structure construction, for representing local sequence and structural information on protein-protein interfaces. Finally, our proposed properties can be effectively applied to identify docking poses, as well as existing energy items.

### Physicochemical property

We can use six physicochemical properties [48, 49] to extract protein features, since one protein can be represented by a vector of physicochemical property. These physicochemical properties are analyzed as hydrophobicity (H), volumes of side chains of amino acids (VSC), polarity (P1), polarizability (P2), solvent-accessible surface area (SASA) and net charge index of side chains (NCISC) of amino acid, respectively. The physicochemical property values of 20 amino acid types are shown in Table 1. They can be normalized to zero mean and unit standard deviation (SD) as follows:

**Table 1:** Original values of six physicochemical properties for 20 types of amino acids

Amino Acid	H	VSC	P1	P2	SASA	NCISC
A	0.62	27.5	8.1	0.046	1.181	0.007187
C	0.29	44.6	5.5	0.128	1.461	-0.03661
D	-0.9	40	13	0.105	1.587	-0.02382
E	-0.74	62	12.3	0.151	1.862	0.006802
F	1.19	115.5	5.2	0.29	2.228	0.037552
G	0.48	0	9	0	0.881	0.179052
H	-0.4	79	10.4	0.23	2.025	-0.01069
I	1.38	93.5	5.2	0.186	1.81	0.021631
K	-1.5	100	11.3	0.219	2.258	0.017708
L	1.06	93.5	4.9	0.186	1.931	0.051672
M	0.64	94.1	5.7	0.221	2.034	0.002683
N	-0.78	58.7	11.6	0.134	1.655	0.005392
P	0.12	41.9	8	0.131	1.468	0.239531
Q	-0.85	80.7	10.5	0.18	1.932	0.049211
R	-2.53	105	10.5	0.291	2.56	0.043587
S	-0.18	29.3	9.2	0.062	1.298	0.004627
T	-0.05	51.3	8.6	0.108	1.525	0.003352
V	1.08	71.5	5.9	0.14	1.645	0.057004
W	0.81	145.5	5.4	0.409	2.663	0.037977
Y	0.26	117.3	6.2	0.298	2.368	0.023599

1 $$ P_{i,j}^{'} = \frac{P_{i,j}-P_{j}}{S_{j}}; \qquad i=1,2,...,20; j=1,2,...,6  $$

where *P*_*i*,*j*_ is the value of physicochemical property *j* for amino acid type *i*, *P*_*j*_ is the mean over 20 amino acid types of physicochemical property *j*, and *S*_*j*_ is the corresponding standard deviation of physicochemical property *j*.

### Position specific scoring matrix

The protein evolutionary information can be described by Position Specific Scoring Matrix (PSSM), generated by PSI-BLAST [50]. Given a protein, the PSSM information is stored in the *L*×20 matrix (protein length: *L*; amino acid types: 20), calculated as follows:

2 $$ PSSM(i,j) \,=\, \sum_{k=1}^{20} \omega(i,k)\times D(k,j); \qquad i\,=\,1,...,L; j\,=\,1,...,20  $$

where *ω*(*i*,*k*) is the frequency of amino acid type *k* at the position *i*, and *D*(*k*,*j*) is the value of Dayhoff’s mutation matrix (substitution matrix) [51] between amino acid types of *k* and *j*.

These PSSM elements can be normalized in a range of [0,1] using the min-max normalization as follows:

3 $$ \begin{aligned} PSSM^{'}(i,j) &= \frac{PSSM(i,j)-PSSM_{min}}{PSSM_{max}-PSSM_{min}};\\ i&=1,...,L; j=1,...,20 \end{aligned}  $$

where *PSSM*_*max*_ and *PSSM*_*min*_ represent the maximal and minimal elements of PSSM.

### Multi-scale local average block

We utilize Multi-scale Local Average Block (MLAB) algorithm to extract the conserved information of local regions. The original Average Block (AB) algorithm was proposed by Jeong et al. [52]. Different from the original AB algorithm, we use multi-scale size to split the matrix horizontally. The MLAB features can describe the local relationship between target residue and neighboring residues. Given a residue *R*, we denote *R*^−1^,*R*^−2^,...,*R*^−5^ be the five residues before *R* in the sequence, and *R*^+1^,*R*^+2^,...,*R*^+5^ be the five residues after *R* in the sequence. Then, *R*^±1^,*R*^±2^,...,*R*^±5^ are referred to as the ten sequential neighbors.

**Fig. 2:**
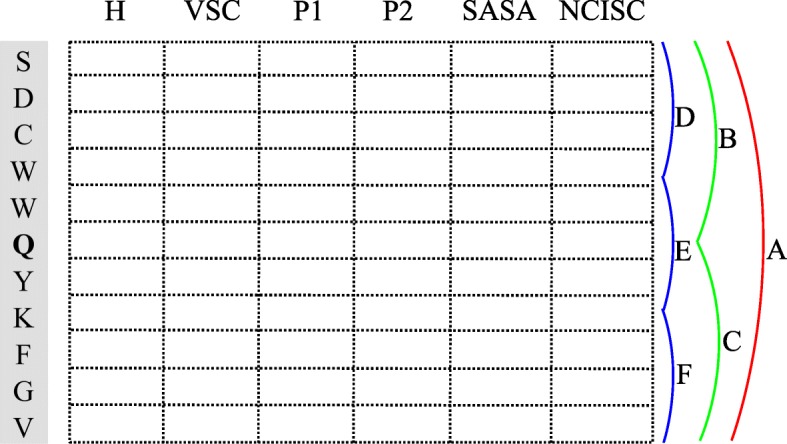
Schematic diagram of Multi-scale Local Average Blocks feature extraction

We split the information of target residue into six local sequential regions with varying composition, via global zone (A), bisection (B and C) and trichotomy (D, E and F). These local regions can describe multiple overlapping continuous and discontinuous interaction patterns, shown in Fig. 2. We calculate the mean of each local block as follows:

4 $$ L(k,j) = \frac{1}{{B_{k}^{L}}} \sum_{i=1}^{{B_{k}^{L}}} {M_{k}^{L}}(i,j); \qquad k=1,...,6; j=1,...,20  $$

where *L*(*k*,*j*) is the mean of *k*-th block in the column *j*, ${B_{k}^{L}}$ is the total number of rows in block *k*, and ${M_{k}^{L}}(i,j)$ is the value of cell in *i*-th row and *j*-th column of block *k*.

### Hexagon structure construction

**Fig. 3:**
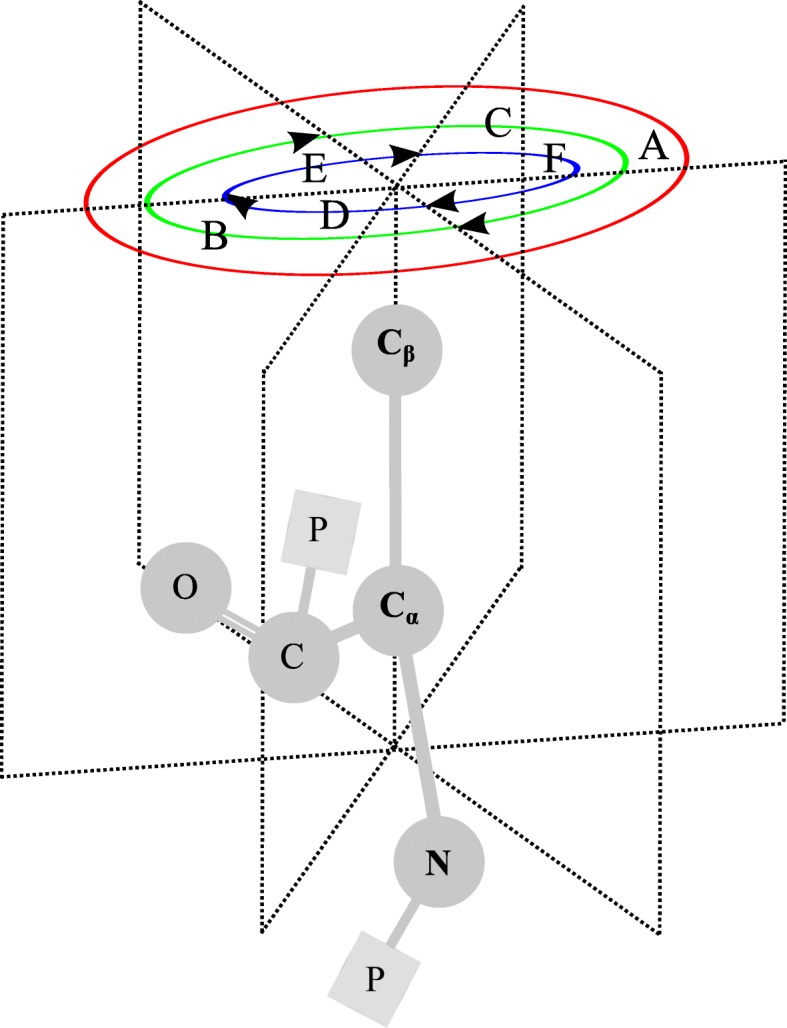
Schematic diagram of Hexagon Structure Construction feature extraction

We build the hexagon structure for each target residue to describe its neighborhood information, as demonstrated in Fig. 3. We assume that *C*_*α*_ is the origin, *C*_*β*_ is along the positive direction of *y*-axis, and *N* is on the *x*-*y* plane where *x* is positive. The 3D space is partitioned along *y*-axis into six equal subspaces by three planes, and the angle between any two planes is 60^∘^. Given a residue *R*, we locate nearest non-local *C*_*α*_ to *C*_*α*_ of residue *R* within a certain distance in each subspace. Here, we say a residue is non-local to residue *R* if and only if it is separated by at least three residues from residue *R* in sequence. We call these six residues as spatial neighbors of residue *R*, denoted as ${H_{R}^{1}}$, ${H_{R}^{2}}$,..., ${H_{R}^{6}}$.

We split the hexagon structure of target residue into six local spatial regions with varying composition, via global zone (A), bisection (B and C) and trichotomy (D, E and F). We calculate the mean of each local space as follows:

5 $$ H(k,j) = \frac{1}{{B_{k}^{H}}} \sum_{i=1}^{{B_{k}^{H}}} {M_{k}^{H}}(i,j); \qquad k=1,...,6; j=1,...,20  $$

where *H*(*k*,*j*) is the mean of *k*-th space in the column *j*, ${B_{k}^{H}}$ is the total number of rows in space *k*, and ${M_{k}^{H}}(i,j)$ is the value of cell in *i*-th row and *j*-th column of space *k*.

### Extracting interface residues

The above proposed features can be effectively applied to extract protein-protein interface residues and identify docking poses, as well as existing energy items. The energy items are listed as follows:

- amino acid contact energy – amino acid probabilities of interface residues [53].
- secondary structure contact energy – secondary structure probabilities of interface residues [53].
- structural neighborhood energy – probability of structural neighboring property on interface [54].
- dihedral angle energy – statistical analysis of dihedral angle correlation on interface [55].
- *π*- *π* interaction energy – geometrical property on *π*- *π* interaction [55].
- multi-scale local average block on protein 1D sequence.
- hexagon structure construction on protein 3D structure.

We use a trained support vector regression (SVR) model to rank docking poses, and then report the top-ranking poses with lowest energy values [56–58]. For the training set, we use *I*_*rmsd*_ (rmsd value between predicted interfaces and native complexes) as the response values for all configurations of each pair of proteins, and the above energy items can be regarded as seven groups of features for each pose. Some configurations with the lowest predicted response values can be reported as the final result on the testing set. For a given pair of proteins, we use the trained SVR model to select top 10 predictions with lowest energy values.

## Results

In this section, we compare our method to many existing methods for identifying protein-protein interfaces. Experiments show that our method performs better than some state-of-the-art methods on Benchmark v4.0 and the CAPRI targets, based on the prediction quality improved in terms of CAPRI evaluation criteria.

### Evaluation criteria

A complex may contain several subunits and multiple binding interfaces. Each binding interface in a complex occurs in a pair of subunits. Two residues between a pair of subunits are called interface residues, if any two atoms, one from each residue, interact. By interacting, the distance between two atoms from a pair of different residues is less than 6Å.

According to CAPRI evaluation criteria [45], three evaluation measures are commonly used in protein-protein interface prediction. A pair of residues on different sides of interface is considered to be in contact if any of their atoms are within 6Å. One is the fraction of native contacts *F*_*nat*_, defined as the number of correct residue-residue contacts in the predicted configuration divided by the number of contacts in the native complex. The other is the fraction of non-native contacts *F*_*non*−*nat*_, defined as the number of incorrect residues-residue contacts in the predicted configuration divided by the total number of contacts in that predicted pose. The third is root-mean-square deviation of interface *I*_*rmsd*_, defined as rmsd value between all backbone atoms of interfaces in predicted pose and in native complex, after two interfaces are superimposed.

The CAPRI evaluation use different cutoffs on these three measures to assign predicted poses into four quality classes: Incorrect (*F*_*nat*_<10*%* or *I*_*rmsd*_>4.0Å), Acceptable (10*%*<=*F*_*nat*_<30*%* and 2.0Å <*I*_*rmsd*_<=4.0Å), Medium (30*%*<=*F*_*nat*_<50*%* and 1.0Å <*I*_*rmsd*_<=2.0Å), or High (*F*_*nat*_>=50*%* and *I*_*rmsd*_<=1.0Å).

### Statistical analysis

We analyze different regression models and evaluate the performance of energy items on CAPRI [45]. CAPRI is a community-wide experiment to assess the capacity of docking methods.

#### Assessment of regression model

**Fig. 4:**
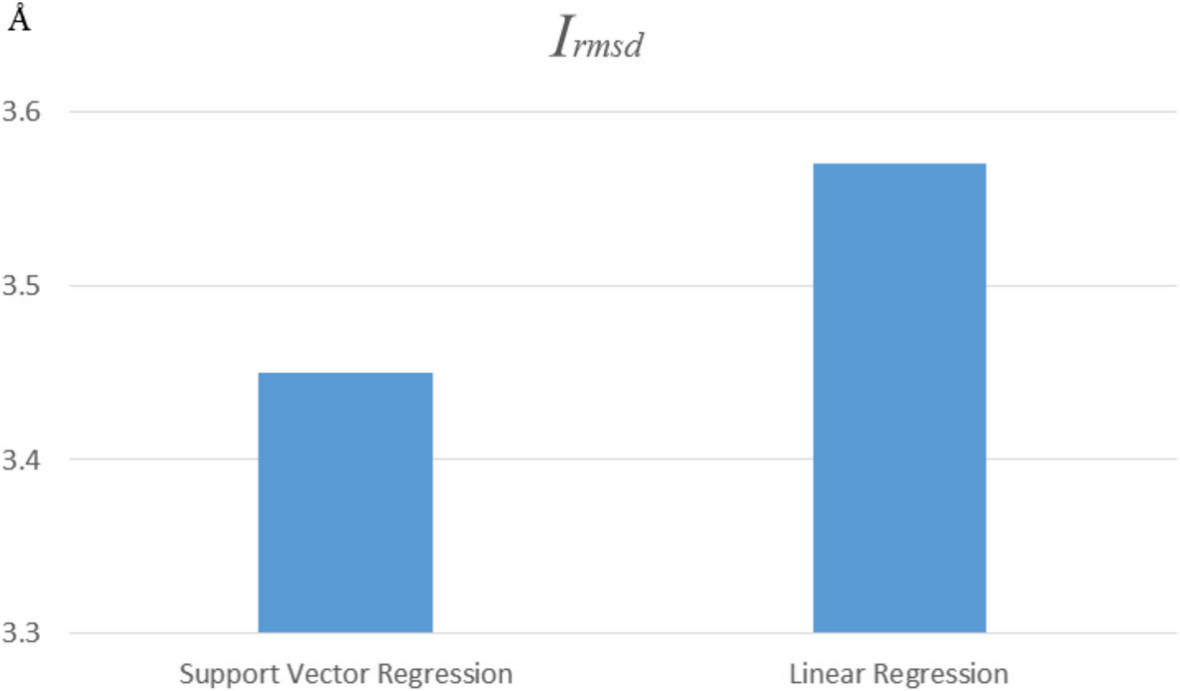
Performance of different regression models on CAPRI

To assess the effectiveness of regression model, we analyze the performance of Support Vector Regression [59] and Linear Regression [60] with same energy items on CAPRI, and the results are shown in Fig. 4. The average *I*_*rmsd*_ value for cases by Support Vector Regression is 3.45Å. The average *I*_*rmsd*_ value for cases by Linear Regression is 3.57Å. It confirms our hypothesis that Support Vector Regression can accurately identify the protein-protein interface.

#### Assessment of energy items

**Fig. 5:**
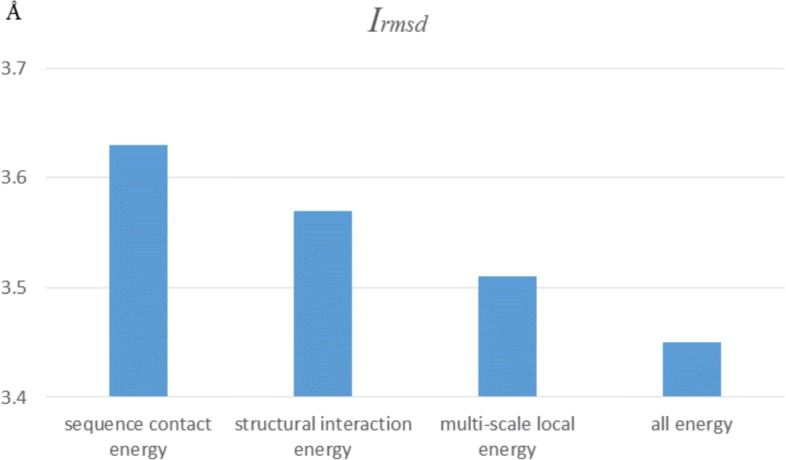
Performance of different energy items on CAPRI

To assess the effectiveness of energy items, we analyze the performance of different cases on CAPRI. We re-evaluate configurations selected by different energy items, and the results are shown in Fig. 5. The average *I*_*rmsd*_ value for cases with sequence contact energy (amino acid contact energy, secondary structure contact energy) is 3.63Å. The average *I*_*rmsd*_ value for cases with structural interaction energy (structural neighborhood energy, dihedral angle energy, *π*- *π* interaction energy) is 3.57Å. The average *I*_*rmsd*_ value for cases with multi-scale local energy (multi-scale local average block on protein 1D sequence, hexagon structure construction on protein 3D structure) is 3.51Å. Average *I*_*rmsd*_ values for these cases are less than that for cases with all energy items (3.45Å). It confirms our hypothesis that the multi-scale local representations on sequence and structural information are the important factors to consider in the protein-protein interface prediction.

### Docking validation

We evaluate the performance of our method on the protein-protein complexes in Benchmark 4.0 [46]. All targets in Benchmark 4.0 are classified into three categories: rigid-body (easy) cases, medium difficult cases and difficult cases, according to the magnitude of conformational change after binding. Our method is compared to SnapDock [38], InterEvDock [37] and FRODOCK 2.0 [36]. The success rate reports the percentage of cases for which at least one out of top 10 predictions is an acceptable or better solution on CAPRI criteria. The protein-protein docking results of different methods are shown in Table 2. The success rates by our method, FRODOCK 2.0, InterEvDock and SnapDock on Benchmark v4.0 are 41.5*%*, 29.0*%*, 29.4*%* and 37.0*%*, respectively. Our method improves the success rate at least by 4.5*%*.

**Table 2:** The prediction results by our method, FRODOCK 2.0, InterEvDock and SnapDock on Benchmark v4.0

	success rate
FRODOCK 2.0	29.0% (51/176)
InterEvDock	29.4% (25/85)
SnapDock	37.0% (57/154)
Our Method	41.5% (73/176)

### Protein-protein interface prediction

In this study, we compare our predicted interfaces with ZRANK [40, 41] and FiberDock(external tool) [28], and also with ClusPro [42]. We consider 79 complexes from Dockground [61] as the training set. In order to avoid over-fitting, we exclude complexes sharing more than 30*%* identity with cases in testing set. The average *I*_*rmsd*_ value is 1.49Å, and the overall *F*_*nat*_ and *F*_*non*−*nat*_ values are 85*%* and 16*%*.

#### Evaluation on benchmark v4.0

On Benchmark v4.0, our method achieves average *I*_*rmsd*_ value of 3.28Å and overall *F*_*nat*_ value of 63*%*, which improves upon *I*_*rmsd*_ of 3.89Å and *F*_*nat*_ of 49*%* for ZRANK, and *I*_*rmsd*_ of 3.99Å and *F*_*nat*_ of 46*%* for ClusPro. Results are shown in Table 3. The complexes are classified into three categories, according to the magnitude of conformational change after binding. In rigid-body group, our method achieves average *I*_*rmsd*_ value of 2.86Å and overall *F*_*nat*_ value of 69*%*, which improves upon *I*_*rmsd*_ of 3.31Å and *F*_*nat*_ of 56*%* for ZRANK, and *I*_*rmsd*_ of 3.33Å and *F*_*nat*_ of 55*%* for ClusPro. In medium difficulty group, our method achieves average *I*_*rmsd*_ value of 3.35Å and overall *F*_*nat*_ value of 59*%*, which improves upon *I*_*rmsd*_ of 4.46Å and *F*_*nat*_ of 39*%* for ZRANK, and *I*_*rmsd*_ of 4.71Å and *F*_*nat*_ of 30*%* for ClusPro. In difficulty group, our method achieves average *I*_*rmsd*_ value of 5.39Å and overall *F*_*nat*_ value of 36*%*, which improves upon *I*_*rmsd*_ of 6.18Å and *F*_*nat*_ of 28*%* for ZRANK, and *I*_*rmsd*_ of 6.53Å and *F*_*nat*_ of 21*%* for ClusPro.

**Table 3:** The prediction results by our method, ZRANK+FiberDock and ClusPro on Benchmark v4.0

Subset^a^	No. of cases	Our Method			ZRANK+FiberDock			ClusPro		
		*I*_*rmsd*_	*F*_*nat*_	*F*_*non*−*nat*_	*I*_*rmsd*_	*F*_*nat*_	*F*_*non*−*nat*_	*I*_*rmsd*_	*F*_*nat*_	*F*_*non*−*nat*_
Rigid	123	2.86	69%	35%	3.31	56%	49%	3.33	55%	51%
Medium	29	3.35	59%	39%	4.46	39%	59%	4.71	30%	69%
Difficult	24	5.39	36%	58%	6.18	28%	67%	6.53	21%	77%
Overall	176	3.28	63%	39%	3.89	49%	53%	3.99	46%	58%

^a^Subset is based on the magnitude of conformational change after binding

#### Evaluation on Capri

We evaluate protein-protein interface prediction by our method, ZRANK and ClusPro on CAPRI. On 35 CAPRI targets, our method achieves average *I*_*rmsd*_ value of 3.45Å and overall *F*_*nat*_ value of 46*%*, which improves upon *I*_*rmsd*_ of 4.18Å and *F*_*nat*_ of 40*%* for ZRANK, and *I*_*rmsd*_ of 5.12Å and *F*_*nat*_ of 32*%* for ClusPro. Our method predicts 9 incorrect, 12 acceptable, 12 medium, 2 high quality results. ZRANK+FiberDock predicts 14 incorrect, 7 acceptable, 7 medium, 7 high quality results. ClusPro predicts 13 incorrect, 11 acceptable, 8 medium, 3 high quality results.

### Binding sites identification

Some existing methods use machine learning and statistical approaches to predict binding sites. Each comparison with an existing method is performed using the test data by the compared method in the literature.

#### Comparison to metaPPI, meta-PPISP and pPI-Pred

In this experiment, the test data in metaPPI [20] is used to predict binding sites. The data consists of 41 complexes, divided into two categories: enzyme-inhibitor (EI) and others. The overall *F*_*nat*_ and *F*_*non*−*nat*_ values for each prediction method are shown in Table 4. The overall *F*_*nat*_ values for our method, metaPPI, meta-PPISP and PPI-Pred achieve 62*%*, 28*%*, 38*%* and 38*%*, respectively. The overall *F*_*non*−*nat*_ values for these four methods achieve 34*%*, 51*%*, 54*%* and 64*%*, respectively. Our method improves the overall *F*_*nat*_ value by at least 24*%*. The average sizes of predicted interface residues for our method, metaPPI, meta-PPISP and PPI-Pred are 22.1, 13.2, 18.2 and 27.8, while the average size of actual interface residues is 22.7. The number of residues predicted correctly for these four methods are 12.9, 5.5, 7.5 and 8.2.

**Table 4:** Comparison to metaPPI, meta-PPISP and PPI-Pred

Type	Our Method		metaPPI		meta-PPISP		PPI-Pred	
	*F*_*nat*_	*F*_*non*−*nat*_	*F*_*nat*_	*F*_*non*−*nat*_	*F*_*nat*_	*F*_*non*−*nat*_	*F*_*nat*_	*F*_*non*−*nat*_
E-I^a^	65%	23%	37%	39%	55%	44%	47%	54%
others	59%	42%	22%	59%	26%	61%	31%	71%
Overall	62%	34%	28%	51%	38%	54%	38%	64%

^a^*E-I* is the type of enzyme-inhibitor

#### Comparison to proMate and pINUP

Our method is compared to ProMate and PINUP. The test data is originally used by ProMate [16], including 57 unbound proteins and their complexes. The results are reported in Table 5. The overall *F*_*nat*_ values for our method, PINUP and ProMate achieve 60*%*, 42*%* and 13*%*, respectively. The overall *F*_*non*−*nat*_ values for these three methods achieve 45*%*, 55*%* and 47*%*, respectively. Our method improves the overall *F*_*nat*_ value by at least 19*%*. The average sizes of predicted interface residues for our method, PINUP and ProMate are 25.6, 19.0 and 5.4, while the average size of actual interface residues is 22.6. The number of residues predicted correctly for these three methods are 12.6, 8.3 and 2.7.

**Table 5:** Comparison to PINUP and ProMate

	Our Method		PINUP		ProMate	
	*F*_*nat*_	*F*_*non*−*nat*_	*F*_*nat*_	*F*_*non*−*nat*_	*F*_*nat*_	*F*_*non*−*nat*_
Overall	60%	45%	42%	55%	13%	47%

### Case study

We evaluate interface prediction of our method on two different cases.

#### Interface prediction on sK/RR interaction

**Fig. 6:**
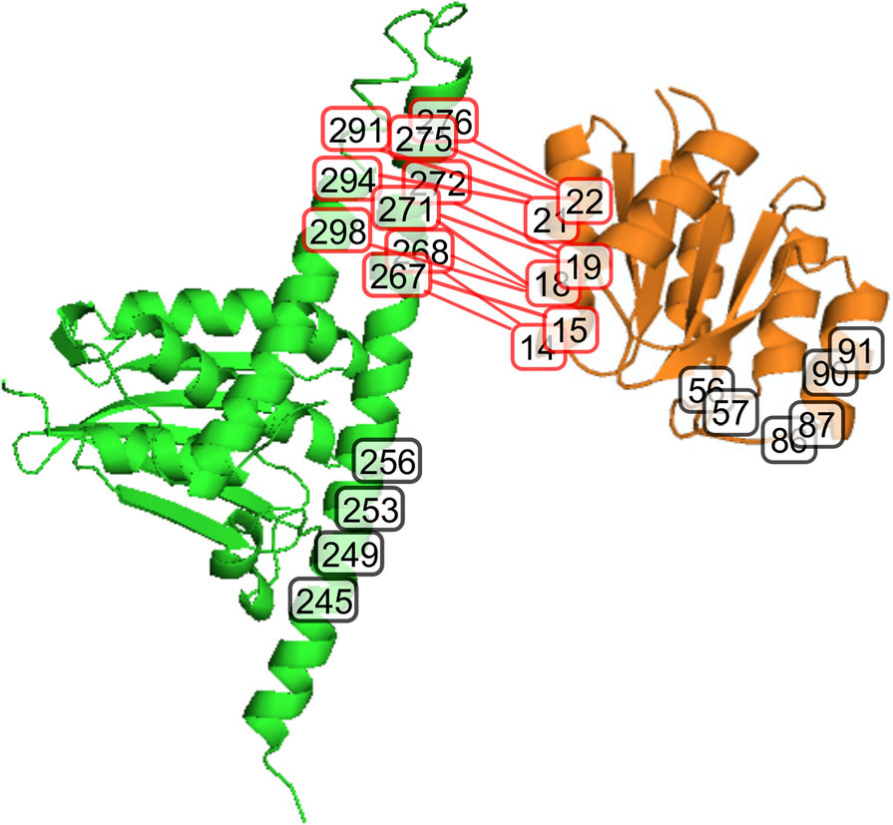
Our method detects the binding residues on SK/RR interaction. Interface residues are described in red boxes and non-interface residues are described in black boxes

We study HisKA domain of sensor histidine kinase (PF00512) and its partner response regulator domain (PF00072) in Pfam database [62]. Interface identification can be tested by using structural representatives of HisKA domain of SK (HK853; PDB ID code 2C2A chain A) and of RR domain (Spo0F; PDB ID code 1PEY chain A), as well as co-crystal structure of Spo0F in complex with Spo0B (PDB ID code 1F51 chain A:E). We analyze 25 interacting residues, involving 13 SK positions and 12 RR positions. For HK853, predicted interface residues being part of interface are 267, 268, 271, 272, 275, 276, 291, 294 and 298, as indicated by red boxes in Fig. 6. Predicted interface residues of SK belonging to non-interface are 245, 249, 253 and 256. For Spo0F, predicted interface residues being part of interface are 14, 15, 18, 19, 21 and 22, as indicated by red boxes in Fig. 6. Predicted interface residues of RR belonging to non-interface are 56, 57, 86, 87, 90 and 91.

#### Interface prediction on spirulina platensis

**Fig. 7:**
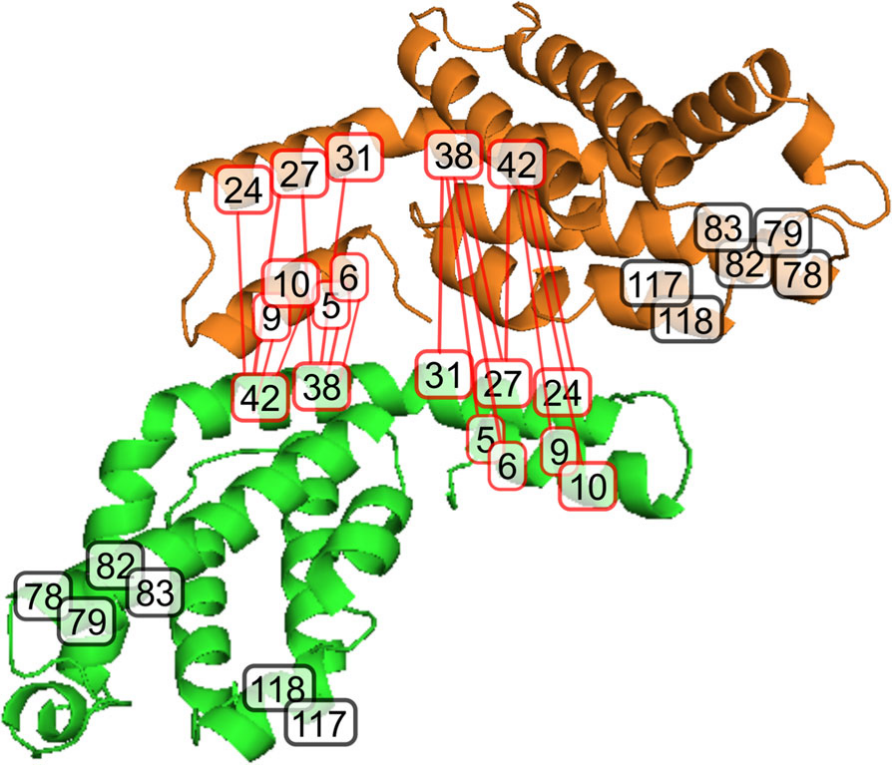
Our method detects the binding residues on spirulina platensis. Interface residues are described in red boxes and non-interface residues are described in black boxes

We study spirulina platensis *α*-subunit (PDB ID code 1GH0 chain A) and *β*-subunit (PDB ID code 1GH0 chain B). We analyze 30 interacting residues, involving 15 *α*-subunit positions and 15 *β*-subunit positions. For *α*-subunit, predicted interface residues being part of interface are 5, 6, 9, 10, 24, 27, 31, 38 and 42, as indicated by red boxes in Fig. 7. Predicted interface residues of *α*-subunit belonging to non-interface are 78, 79, 82, 83, 117 and 118. For *β*-subunit, predicted interface residues being part of interface are 5, 6, 9, 10, 24, 27, 31, 38 and 42, as indicated by red boxes in Fig. 7. Predicted interface residues of *β*-subunit belonging to non-interface are 78, 79, 82, 83, 117 and 118.

## Discussion

Lots of protein-protein identification approaches are based on analyzing some different features, such as sequence and structural properties, as well as other physicochemical properties. Most of the features only describe the property of current interacting residues, but cannot represent real situation well, thus are insufficient to predict interface residues with high accuracy. Although many computational methods have been used to predict protein-protein interfaces, the effectiveness and robustness of previous prediction models can still be improved. Main improvements of our proposed method come from adopting the effective feature extraction models that can capture useful protein information. All results demonstrate that our method is a valuable technological tool for identifying protein-protein interface.

## Conclusions

We identify two new features: multi-scale local average block and hexagon structure construction. Given a pair of proteins, we use the trained SVR model to select best poses. From experimental results, the prediction ability of our method is better than that of other existing state-of-the-art approaches. It demonstrates that our proposed method is a very promising and useful support tool for future proteomics research. In the future work, we will extend our method to predict important special complexes.

## Data Availability

The datasets used or analysed during the current study are available from https://github.com/guofeieileen/binder.

## Abbreviations

CAPRI: Critical Assessment of PRediction of Interactions
EI: enzyme-inhibitor
H: Hydrophobicity
MLAB: Multi-scale Local Average Block
NCISC: Net Charge Index of Side Chains of amino acid
P1: Polarity
P2: Polarizability
PSSM: Position Specific Scoring Matrix
SASA: Solvent-Accessible Surface Area
SVR: Support Vector Regression
VSC: Volumes of Side Chains of amino acids
